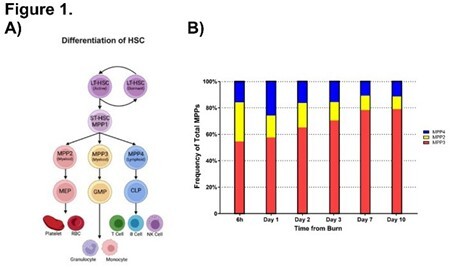# 15 Burn Injury Reveals Myeloid Priming at the Progenitor Level in the Murine Bone Marrow Niche

**DOI:** 10.1093/jbcr/irae036.015

**Published:** 2024-04-17

**Authors:** Ryan M Johnson, Kevin E Galicia, Huashan Wang, Mashkoor A Choudhry, John Kubasiak

**Affiliations:** Loyola University Chicago, Chicago, Illinois; Loyola University, Maywood, Illinois; Loyola University Chicago, Chicago, Illinois; Loyola University, Maywood, Illinois; Loyola University Chicago, Chicago, Illinois; Loyola University, Maywood, Illinois; Loyola University Chicago, Chicago, Illinois; Loyola University, Maywood, Illinois; Loyola University Chicago, Chicago, Illinois; Loyola University, Maywood, Illinois

## Abstract

**Introduction:**

Hematopoiesis is the process of forming blood cells from hematopoietic stem cells (HSCs), ultimately generating erythroid, myeloid, and lymphoid lineages (Fig 1A). Key regulators in differentiation of HSCs include transcription factors GATA-1 (erythroid), PU.1 (myeloid/lymphoid; known to oppose GATA-1), GATA-3 (lymphoid cell lines), and RUNX1 (essential for HSC formation and hematopoietic balance). In particular, burn injuries have been shown to lead to a prevalence of myeloid cells in the body, which is associated with unfavorable patient outcomes, an increased risk of shock, susceptibility to infections, and anemia. Understanding the factors governing the immune and inflammatory response within the bone marrow environment following burn injuries is crucial for targeted therapies and improved patient outcomes.

**Methods:**

C57BL/6 mice were divided into two groups: one receiving burn injuries (12% TBSA to dorsal surface after exposure to 85°C water immersion for 7 seconds) and one with a sham procedure (n=12 per group). Each group was sacrificed at 6 hours, and at days 1, 2, 3, 7, and 10 post-burn. Bone marrow samples were collected from hindlimbs for flow cytometry to examine HSC populations. The above burn injury and bone marrow extraction protocol was utilized on another burn and sham group of C57BL/6 mice (n=12 per group) before sacrifice at days 1, 3, and 7 with subsequent lineage negative (CD5, CD11b, CD45R (B220), Anti-Gr-1 (Ly-6G/C), 7-4, and Ter-119 negative) cell isolation. RNA from these cells was isolated, purity tested, and used for qPCR, with gene expression normalized to GAPDH and presented as fold changes compared to control levels.

**Results:**

Flow cytometry revealed the proportion of Multipotent Progenitor Cell 3 (MPP3; destined to be myeloid/granulocytic) increased from 54% to 78% over 10 days post-burn (Fig 1B). qPCR was used to analyze the expression of transcription factors PU.1, RUNX1, GATA1, and GATA3 in both burn and sham control mice. PU.1 was decreased in burn mice on day 1 (p = 0.0002) but increased by day 7 (p < 0.01) (Fig 2A). RUNX1 was decreased in burn mice on days 1 and 3 (p < 0.01) (Fig 2B). GATA1 decreased by day 7 (p < 0.05) (Fig 2C), and GATA3 decreased on days 3 and 7 (p < 0.05) (Fig 2D).

**Conclusions:**

These findings suggest dynamic alterations in the expression profiles of these transcription factors in response to burn injury, with significant changes occurring at various time points. Of specific concern is the shutdown of regulators for lymphopoiesis, and the upregulation of myelopoiesis regulators. These changes may result in the increased risk of sepsis seen in large burn injuries. Further work is necessary to understand the dynamic regulation of the host immune response at the level of the HSPC.

**Applicability of Research to Practice:**

Detailing the immune response to burn injury could lead to novel therapies designed to drive the immune system in a more favorable direction for patient recovery.